# A Protective Role for ELR+ Chemokines during Acute Viral Encephalomyelitis

**DOI:** 10.1371/journal.ppat.1000648

**Published:** 2009-11-06

**Authors:** Martin P. Hosking, Liping Liu, Richard M. Ransohoff, Thomas E. Lane

**Affiliations:** 1 Department of Molecular Biology and Biochemistry, University of California, Irvine, California, United States of America; 2 Neuroinflammation Research Center, Department of Neurosciences, Cleveland Clinic, Cleveland, Ohio, United States of America; 3 Institute for Immunology, Infectious Diseases, and Vaccines, University of California, Irvine, California, United States of America; Duke University Medical Center, United States of America

## Abstract

The functional role of ELR-positive CXC chemokines in host defense during acute viral-induced encephalomyelitis was determined. Inoculation of the neurotropic JHM strain of mouse hepatitis virus (JHMV) into the central nervous system (CNS) of mice resulted in the rapid mobilization of PMNs expressing the chemokine receptor CXCR2 into the blood. Migration of PMNs to the CNS coincided with increased expression of transcripts specific for the CXCR2 ELR-positive chemokine ligands CXCL1, CXCL2, and CXCL5 within the brain. Treatment of JHMV-infected mice with anti-CXCR2 blocking antibody reduced PMN trafficking into the CNS by >95%, dampened MMP-9 activity, and abrogated blood-brain-barrier (BBB) breakdown. Correspondingly, CXCR2 neutralization resulted in diminished infiltration of virus-specific T cells, an inability to control viral replication within the brain, and 100% mortality. Blocking CXCR2 signaling did not impair the generation of virus-specific T cells, indicating that CXCR2 is not required to tailor anti-JHMV T cell responses. Evaluation of mice in which CXCR2 is genetically silenced (*CXCR2−/−* mice) confirmed that PMNs neither expressed CXCR2 nor migrated in response to ligands CXCL1, CXCL2, or CXCL5 in an *in vitro* chemotaxis assay. Moreover, JHMV infection of *CXCR2−/−* mice resulted in an approximate 60% reduction of PMN migration into the CNS, yet these mice survived infection and controlled viral replication within the brain. Treatment of JHMV-infected *CXCR2−/−* mice with anti-CXCR2 antibody did not modulate PMN migration nor alter viral clearance or mortality, indicating the existence of compensatory mechanisms that facilitate sufficient migration of PMNs into the CNS in the absence of CXCR2. Collectively, these findings highlight a previously unappreciated role for ELR-positive chemokines in enhancing host defense during acute viral infections of the CNS.

## Author Summary

Consequences of viral infection of the central nervous system (CNS) can range from encephalitis and paralytic poliomyelitis to relatively benign infections with limited clinical outcomes. The localized expression of proinflammatory chemokines within the CNS in response to viral infection has been shown to be important in host defense by attracting antigen-specific lymphocytes from the microvasculature into the parenchyma that control and eventually eliminate the replicating pathogen. However, the relationship between chemokine expression and recruitment of myeloid cells, *e.g.* neutrophils, to the CNS following infection with a neurotropic virus is not well characterized. Emerging evidence has indicated that the mobilization of neutrophils into the blood and recruitment to the CNS following microbial infection or injury contributes to permeabilization of the blood-brain-barrier that subsequently allows entry of inflammatory leukocytes. Therefore, we have defined the chemokines involved in promoting the directional migration of neutrophils to the CNS in response to viral infection. Using the neurotropic JHM strain of mouse hepatitis virus (JHMV) as a model of acute viral encephalomyelitis, we demonstrate a previously unappreciated role for members of the ELR-positive CXC chemokine family in host defense by attracting PMNs bearing the receptor CXCR2 to the CNS in response to viral infection.

## Introduction

Inoculation of the neurotropic JHMV strain of mouse hepatitis virus (a positive-strand RNA virus and member of the *Coronaviridae* family) into the CNS of susceptible strains of mice results in an acute encephalomyelitis, characterized by wide spread infection and replication within astrocytes, microglia, and oligodendrocytes, while relatively sparing neurons [1]. Mechanisms associated with control of viral growth are dictated by the infected host cell. Astrocytes and microglia are susceptible to perforin-mediated lysis by cytotoxic T lymphocytes [2], whereas IFN-γ suppresses viral replication within oligodendrocytes [3]. Although a robust cell-mediated immune response occurs during acute disease, sterilizing immunity is not achieved, resulting in viral persistence [4]. While virus-specific CD8^+^ T cells are retained within the CNS of persistently infected mice and lytic activity is muted, these cells retain the capacity to secrete IFN-γ that limits viral replication in oligodendrocytes [3], [5]–[7]. Histological features associated with viral persistence include the development of an immune-mediated demyelinating disease similar to the human demyelinating disease multiple sclerosis (MS), with both T cells and macrophages being important in amplifying disease severity by contributing to myelin damage [8],[9].

Chemokines are rapidly secreted within the CNS in response to JHMV infection and contribute to host defense [10]–[14] and disease progression [10], [15]–[17]. The ELR+ (glutamic acid – leucine – arginine) CXC chemokines CXCL1 and CXCL2 are up-regulated within the brains of JHMV-infected mice [11],[18],[19], yet little is known regarding their biological significance or cellular targets. CXCL1 and CXCL2 are potent chemoattractants for PMNs, binding and signaling through their receptor CXCR2 [20]–[22]. Moreover, PMNs have been shown to enhance CNS inflammation by disrupting blood brain barrier (BBB) integrity in animal models of spinal cord injury (SCI) [23],[24], autoimmune demyelination [25], and JHMV-induced encephalomyelitis [26]. In addition, blocking or silencing of CXCR2 signaling mutes inflammation and tissue damage in mouse models in which PMN infiltration is critical to disease initiation, including SCI [23], inflammatory demyelination [25], bacterial infection of the CNS [27], and viral infection or injury to the lung [28]–[32]. With regards to JHMV infection, depletion of PMNs increases mortality due to abrogated BBB permeabilization and subsequent diminished T cell infiltration into the CNS, however the relationship between CXCR2 signaling and PMN migration during viral infection of the CNS has yet to be determined.

The present study was undertaken to characterize the functional role of ELR+ chemokines in either host defense or disease following viral infection of the CNS. Using JHMV infection as a model of viral-induced encephalomyelitis, we demonstrate a protective role for ELR-positive chemokines in promoting PMN migration to the CNS and subsequent BBB degradation, facilitating the entry of T lymphocytes and control of viral replication.

## Results

### CXCR2-positive PMNs are rapidly recruited to the CNS in response to JHMV infection

**Figure 1:**
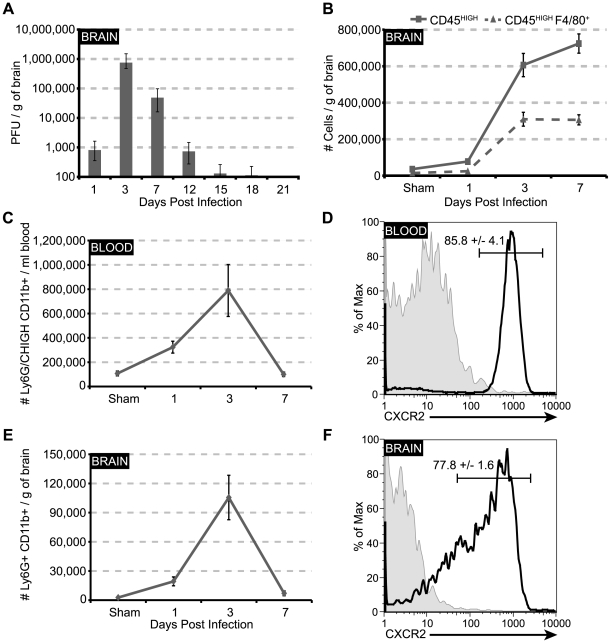
CXCR2+ neutrophils peak early during JHMV infection. C57BL/6 mice were i.c. infected with 500 PFU JMHV; blood and brains were removed at defined times p.i. for viral titer or flow cytometric analysis. (**A**) Following infection JHMV rapidly replicates within the brains of infected mice, peaking at day 3 p.i. Within the (**C**) blood and (**E**) brains of infected mice, neutrophils peak at day 3 p.i. before quickly returning to sham levels by day 7 p.i. Approximately 80% of these neutrophils within the (**D**) blood and (**F**) brain are CXCR2-positive. The infiltration kinetics of neutrophils are unique to this subset of cells, as (**B**) both total inflammatory cells (CD45^high^) and macrophages (CD45^high^F4/80^+^) continue to infiltrate or maintain their levels from day 3 until day 7 p.i. Data are representative of 2 independent experiments with a minimum of 3 mice per time point.

**Figure 2:**
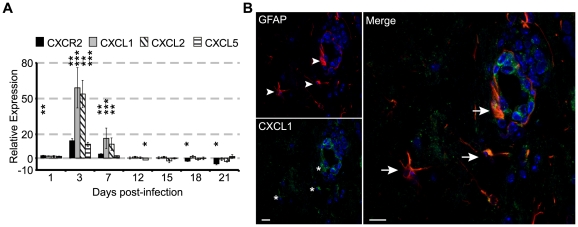
CXCR2 and ELR^+^ chemokines are expressed in the brain during acute JHMV infection. C57BL/6 mice were i.c. infected with 500 PFU JMHV and brains removed at defined times p.i. to determine the expression of transcripts specific for CXCR2, CXCL1,-2, and -5 using semi-quantitative PCR analysis (**A**). Data represent the fold-induction compared to sham-infected mice (n = 3–5 per time point, *p<0.05, **p<0.01, ***p<0.001). (**B**) Representative immunofluorescence staining of brain at day 3 p.i. for astrocytes (GFAP, red staining indicated by arrowheads) and CXCL1 (green staining, indicated by asterisks). Dual-positive cells (indicated by arrows within the merged image) were present surrounding the vessel wall as well as within the parenchyma. CXCL1 staining was enriched within the hippocampus at day 3 p.i. Bar  = 10 µm.

To evaluate the early immune response following JHMV infection, C57BL/6 mice were intracerebrally (i.c.) inoculated with JHMV, and the accumulation of neutrophils within the blood and the CNS was monitored. Within the brain, JHMV titers peaked at day 3 p.i. and gradually declined to below the level of detection (∼100 PFU/g) by day 15 p.i. (**Figure 1A**). Elevated numbers of neutrophils were observed within the blood as early as day 1 p.i. PMN peaked at day 3 p.i, before rapidly returning to sham levels by day 7 p.i. (**Figure 1C**). Similarly, neutrophil infiltration into the CNS peaked at day 3 p.i. and subsequently returned to baseline levels by day 7 p.i. (**Figure 1E**). The chemokine receptor CXCR2 was expressed on the majority of PMNs within the blood (**Figure 1D**) and brain (**Figure 1F**), as assessed by flow cytometry. The pattern of neutrophil infiltration into the CNS was unique, as the infiltration of inflammatory leukocytes (CD45^high^) and macrophages (F4/80^+^CD45^high^) steadily increased to day 7 p.i. (**Figure 1B**). As the majority of neutrophils were CXCR2-positive, we next determined the expression pattern of chemokine ligands capable of binding and signaling through CXCR2 within the brain during JHMV infection. Expression of transcripts for the ELR-positive chemokines CXCL1, CXCL2, and CXCL5, as well as CXCR2, paralleled PMN recruitment into CNS, peaking at day 3 p.i. and returning to baseline levels by day 12 p.i. (**Figure 2A**). Immunostaining for CXCL1 and the astrocyte marker GFAP at day 3 p.i. revealed dual-positive cells located within the parenchyma and associated with the microvasculature (**Figure 2B**). Staining was concentrated within the hippocampal region of the brain. The majority (>80%) of CXCL1-positive cells were astrocytes, as defined by GFAP staining, although endothelial cells also appeared positive, given the enriched CXCL1 signal intensity surrounding the vessel wall (**Figure 2B**). These results are consistent with previous results [11], [33]–[35] that astrocytes, as well as endothelial cells, are capable of producing this CXCR2 ligand. These data indicate that JHMV infection and replication results in regulated expression of ELR+ chemokines and CXCR2 within the CNS that parallels neutrophil mobilization into the blood and migration into the CNS.

### CXCR2 neutralization prevents neutrophil accumulation within the CNS during acute JHMV-induced disease

**Figure 3:**
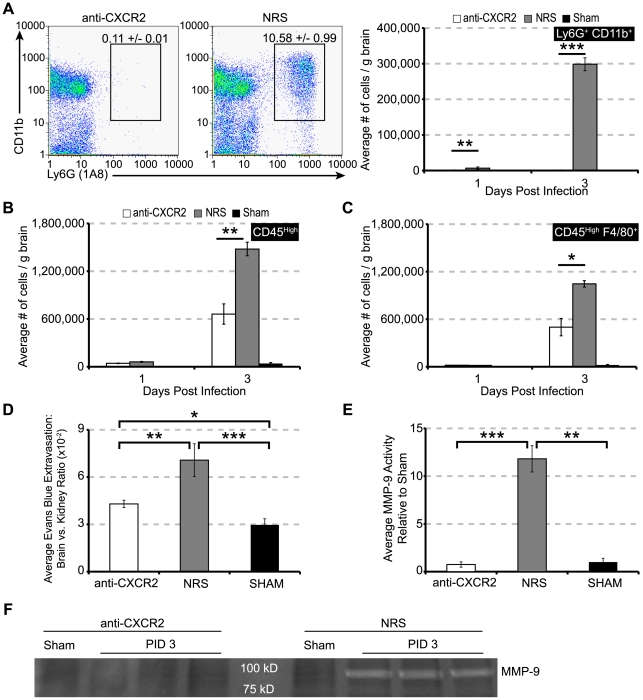
CXCR2 neutralization prevents CNS PMN infiltration and abrogates BBB breakdown. C57BL/6 mice i.c. infected with 500 PFU JMHV were pretreated with CXCR2 neutralizing antiserum or control NRS every other day beginning at day -1 p.i. Brains were removed at days 1 and 3 p.i. to assess cellular infiltration, Evans Blue (EB) extravasation, or MMP activity. CXCR2 neutralization significantly reduced (**A**) the frequency and total numbers of neutrophils within the brain at day 1 and 3 p.i. The infiltration of (**B**) CD45^high^-expressing cells and (**C**) macrophages was also significantly reduced at day 3 p.i. Concomitant with the absence of PMN accumulation, CNS EB extravasation (**D**) was significantly reduced compared to NRS-treated mice, although BBB permeability was greater than sham mice. Additionally, MMP9 activity was undetectable in anti-CXCR2 treated brains compared to NRS-treated mice (**F**). Quantification of MMP9 activity revealed a significant increase in MMP9 activity compared to both sham and infected mice treated with CXCR2 antiserum (**E**). Data in panels ***A*** *–* ***F*** represents 2 independent experiments with a minimum of 3 mice per time point for each experimental group. For panel ***F***, each lane represents an individual mouse. *p<0.05, **p<0.01, ***p<0.001 compared to NRS-treated mice.

To assess the role of CXCR2 during acute viral encephalomyelitis, JHMV infected C57BL/6 mice were treated with neutralizing polyclonal CXCR2 antiserum or control rabbit serum (NRS), and the effect on PMN accumulation within the CNS determined. As shown in **Figure 3A**, representative FACS dot plots revealed an almost complete absence of PMN accumulation at day 3 p.i. within the CNS of mice treated with anti-CXCR2 blocking antibody. Enumeration of total PMN infiltration to the brain showed a significant reduction compared to mice treated with control serum at days 1 (*p*<0.05) and 3 p.i. (*p*<0.01) (**Figure 3A**). CXCR2 neutralization also retarded the accumulation of total inflammatory cells (**Figure 3B**) and macrophages (**Figure 3C**) at day 3 p.i. Further, treatment with CXCR2 antiserum resulted in a significant (*p*<0.05) reduction of neutrophil numbers within the blood at day 3 p.i. (**Figure S1**). Importantly, anti-CXCR2 treatment did not induce complement-mediated lysis or neutrophil depletion from bone-marrow (data not shown). Previously, neutrophils have been deemed partly responsible for the permeabilization of the BBB following JHMV infection [19],[26], therefore, we assessed BBB integrity in the brains of mice treated with either anti-CXCR2 or NRS. Indeed, compared to NRS treatment, CXCR2 neutralization significantly (*p*<0.05) reduced BBB breakdown, as measured by Evans Blue uptake (**Figure 3D**). However, BBB permeability in anti-CXCR2 treated mice was not completely eliminated compared to sham – infected mice (**Figure 3D**). Previous studies indicated that MMP-9 expression by PMN is associated with BBB permeabilization in response to JHMV infection [19],[26]. Consistent with reduced numbers of neutrophils in the brains of anti-CXCR2 treated mice, MMP-9 activity was significantly (*p*<0.001) muted within the brains, whereas detectable levels of enzyme activity were observable within the brains of NRS treated mice (**Figure 3E & 3F**). These data indicate that CXCR2 signaling promotes the directed migration of neutrophils from the periphery to the CNS, thus facilitating the degradation of the BBB in response to JHMV infection.

### CXCR2 neutralization exacerbates mortality, delays viral clearance, and dampens neuro – inflammation

**Figure 4:**
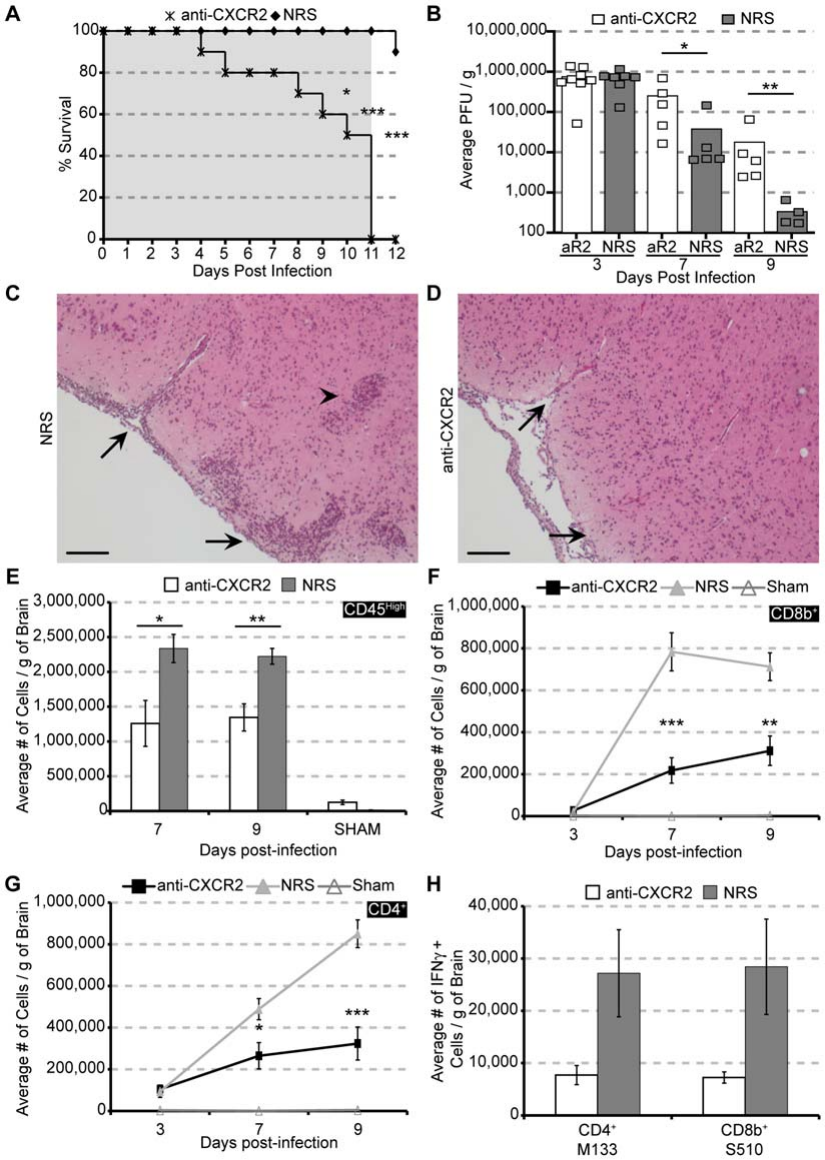
Neutralizing CXCR2 during acute JMHV infection increases mortality, viral burden, and reduces T lymphocyte infiltration. CXCR2 neutralizing antiserum or normal rabbit serum (NRS) was administered every other day from days -1 to 11 p.i. to C57BL/6 mice i.c. infected with 500 PFU of JMHV. The shaded area indicates the treatment window. Treatment with anti-CXCR2 significantly increased mortality (**A**) compared to NRS-treated mice (anti-CXCR2, n = 10; NRS, n = 10), and this was associated with increased viral titers within the brains (**B**) of anti-CXCR2-treated mice compared to control mice. Data presented within panels **A** and **B** are cumulative results from 2 experiments. Representative H&E staining of brains from (**C**) NRS or (**D**) anti-CXCR2 mice at day 9 p.i. reveals a drastic reduction in meningeal (arrows) and parenchymal (arrowhead) inflammation following CXCR2 neutralization. Flow cytometric analysis revealed significantly reduced cellular infiltration of CD45^high^ cells (**E**) and both CD8b^+^ (**F**) and CD4^+^ (**G**) T lymphocyte subsets. In addition, intracellular IFN-γ staining following *ex vivo* stimulation with immunodominant CD4+ (M_133–147_) or CD8+ (S_510–518_) epitopes revealed reduced numbers of virus-specific T cells within the brains of anti-CXCR2-treated mice compared to NRS-treated mice at day 7 p.i. (**H**). Data in panels **E-H** are representative of 2–4 independent experiments with a minimum of 4 mice per time point in each experimental group. *p<0.05, **p<0.01, ***p<0.001 compared to NRS-treated mice. Bars: (**C & D**) 100 µm.

To better assess the functional role of CXCR2 in host defense, JHMV-infected mice were treated with anti-CXCR2 beginning day −1 p.i. and continuing throughout acute disease. Mice began to die at day 4 p.i. and 100% of mice treated with anti-CXCR2 were dead by day 11 p.i., while greater than 90% of infected mice treated with control serum survived until day 12 p.i. (**Figure 4A**). Anti-CXCR2-treated mice were also unable to control viral replication within the brain, as evidenced by the significantly elevated viral titers at days 7 (*p<*0.05) and 9 p.i. (*p<*0.01), compared to mice treated with control serum (**Figure 4B**). Sham – infected mice treated with anti-CXCR2 did not exhibit any signs of morbidity or mortality (data not shown). To further explore the consequences of blocking CXCR2 signaling early following infection, neuroinflammation was assessed by H&E staining of brains from JHMV-infected mice treated with either anti-CXCR2 or control sera at day 9 p.i. Such analysis revealed limited inflammatory cell infiltration, as demonstrated by an overall reduction in the size of the meningeal and perivascular infiltrates (**Figure 4D**), compared to mice treated with control serum (**Figure 4C**). Consistent with the reduced inflammation observed histologically, immunophenotyping the cellular infiltrate in the brain revealed an overall reduction in CD45^high^ cells present within the brains of infected mice treated with anti-CXCR2 (**Figure 4E**). Moreover, blocking CXCR2 reduced CD8^+^ (**Figure 4F**) and CD4^+^ (**Figure 4G**) T cell infiltration, as well as reduced the numbers of virus-specific CD4^+^ and CD8^+^ T cells (**Figure 4H**) within the brains compared to control-treated mice. In contrast, if administration of anti-CXCR2 was delayed until 2 days p.i., mortality, T cell infiltration, and viral burden were unaffected, when compared to control-treated mice (**Figure S2A–D**). Notably, a single treatment of CXCR2 antiserum at day +2 p.i. significantly (*p<*0.01) reduced neutrophil infiltration into the CNS at day 3 p.i. (anti-CXCR2, 8.3×10^3^±1.7×10^3^ cells/g, n = 5) compared to control-treated mice (5.0×10^5^±1.2×10^5^, n = 5) (**Figure S2E**). However, compared to mice that were treated with CXCR2 antiserum beginning at day −1 p.i., the levels of neutrophils within the CNS at day 3 p.i. was approximately 5 times greater [anti-CXCR2, 1.7×10^3^±2.7×10^2^, n = 5; control-treated, 3×10^5^±1.8×10^4^, n = 3) (**Figure 3A**). These findings indicate that CXCR2 signaling is protective; however the period of protection is confined to the earliest days following JHMV infection, coinciding with dramatic mobilization of PMNs from the blood and subsequent migration into the CNS.

### Blocking CXCR2 does not affect generation of virus-specific T cells

**Figure 5:**
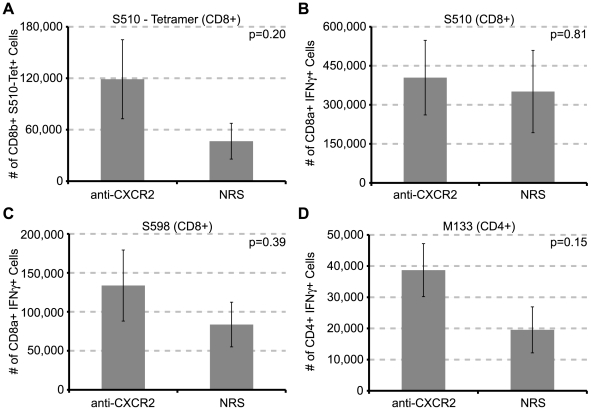
CXCR2 neutralization does not alter the generation of JHMV – specific T cells. CXCR2 neutralizing antiserum or normal rabbit serum (NRS) was administered every other day from days −1 to 5 p.i. to C57BL/6 mice i.c. infected with 500 PFU of JMHV. Cervical lymph nodes were removed on day 7 p.i. Isolated cells were stained with S_510–518_ MHC-I tetramer (**A**) or stimulated *ex vivo* for 6 hours with 5 µM of the immunodominant CD8 epitope S_510–518_ (**B**), or the subdominant CD8 epitope S_598–605_ (**C**), or the immunodominant CD4 epitope M_133–147_ (**D**), and stained for IFN-γ production. Data is representative of two independent experiments with a minimum of 4 mice per experimental group.

**Figure 6:**
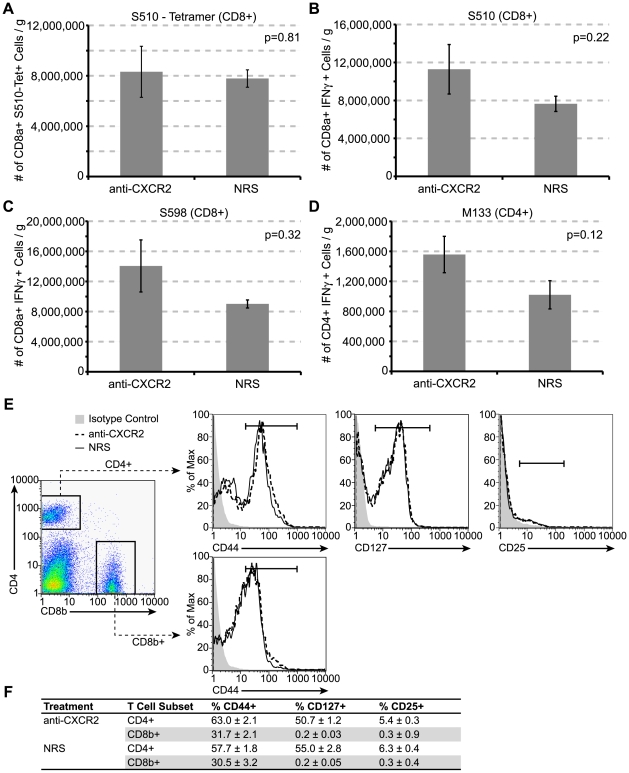
CXCR2 neutralization does not alter the generation of JHMV – specific T cells or total T cell activation within the spleen. CXCR2 neutralizing antiserum or normal rabbit serum (NRS) was administered every other day from days −1 to 5 p.i. to C57BL/6 mice i.p. infected with 2.5×10^5^ PFU of JMHV. Spleens were removed on day 7 p.i. Isolated splenocytes were stained with S_510–518_ MHC-I tetramer (**A**) or stimulated *ex vivo* for 6 hours with 5 µM of the immunodominant CD8 epitope S_510–518_ (**B**), or the subdominant CD8 epitope S_598–605_ (**C**), or the immunodominant CD4 epitope M_133–147_ (**D**), and stained for IFN-γ production. Gated CD4+ or CD8b+ cells (**E**) were also assessed for their expression of the T cell activation markers CD44, CD127, and CD25. CD8b+ cells were negative for CD127 and CD25. The average expression ± SEM of T cell activation markers was tabulated in (**F**, n = 3 anti-CXCR2, n = 5 NRS). Data in panels *A–D* is representative of two independent experiments with a minimum of 4 mice per experimental group. Representative FACS plots are shown in panel *E*.

We next determined if CXCR2 signaling was important in the generation of virus-specific T cells. C57BL/6 mice were treated with either anti-CXCR2 or control serum beginning at day −1 prior to being infected i.c. with JHMV, and the presence of virus-specific T cells within the draining cervical lymph nodes (CLN) [36] was determined at day 7 p.i. by evaluating *ex vivo* responses to defined CD4+ and CD8+ T cell-specific viral epitopes [37]–[39]. Similar numbers of virus – specific CD8+ T cells were generated in mice treated with either anti-CXCR2 or control sera as determined by measuring S_510–518_ MHC class I tetramer – reactive cells (**Figure 5A**) and intracellular staining for IFN-γ within cultured cells pulsed with either the S_510–518_ (**Figure 5B**) or S_598–605_ (**Figure 5C**) peptides. Similarly, there were no differences in the numbers of CD4+ T cells recognizing the M_133–147_ peptide in anti-CXCR2-treated mice compared to control mice (**Figure 5D**). It has been reported that the absence of CXCR2 abrogates peripheral immune responses to pathogens [40]–[46], therefore we also sought to determine whether antiviral responses were muted following CXCR2 neutralization following intraperitoneal (i.p.) challenge with JHMV. C57BL/6 mice were treated with either anti-CXCR2 or control serum beginning at day -1 before being infected i.p. with virus. The presence of virus – specific T cells within the spleen was assessed as described above. Similar frequencies and numbers of virus – specific CD8+ and CD4+ T cells were generated in both control and anti-CXCR2 antiserum treated animals (**Figure 6 A–D**). Moreover, no difference in the expression of the T cell activation markers CD25, CD127, and CD44 was observed upon splenic T cells from mice treated with CXCR2 antiserum (**Figure 6E and F**). Finally, i.p. infection of *CXCR2−/−* mice with JHMV did not affect the generation of virus-specific CD4+ or CD8+ T cells compared to infected *CXCR2+/+* mice (**Figure S3**). Therefore, these data clearly indicate that CXCR2 signaling does not influence the generation or expansion of virus – specific T cells following JHMV infection.

### CXCR2 deficiency attenuates neutrophil infiltration to the CNS without affecting survival or viral clearance following JHMV infection

**Figure 7:**
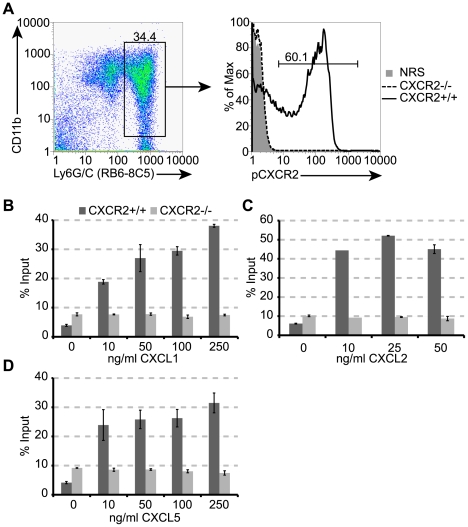
*CXCR2 −/−* neutrophils are unresponsive to CXCR2 ligands. (**A**) Neutrophils, enriched from the bone marrows of *CXCR2+/+* and *CXCR2−/−* mice, were stained with CXCR2 antiserum or control rabbit serum (NRS) and processed for flow cytometric analysis. Neutrophils isolated from *CXCR2−/−* mice were unreactive for CXCR2 antiserum while approximately 60% of the neutrophils from *CXCR2+/+* mice stain positive for CXCR2. Enriched neutrophils were also stimulated with the recombinant chemokines (**B**) CXCL1, (**C**) CXCL2, and (**D**) CXCL5 at the indicated concentrations and allowed to migrate for three hours. Data is representative of two independent experiments.

**Figure 8:**
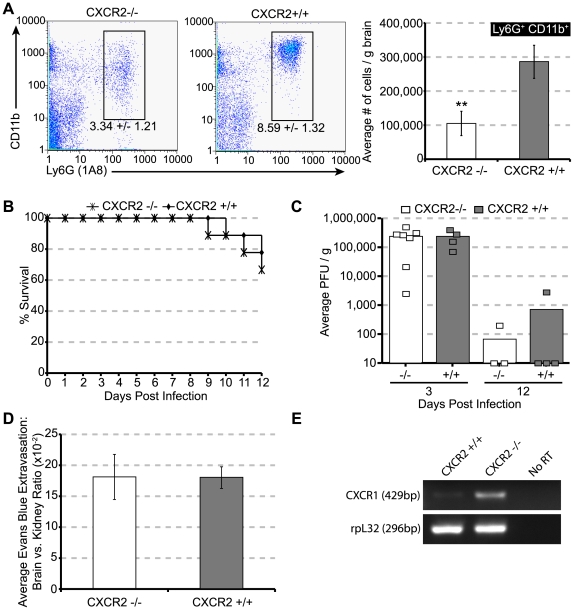
CXCR2 deficiency attenuates neutrophil infiltration but does not affect mortality or viral load. *CXCR2+/+* and *CXCR2−/−* mice were infected i.c. with 500 pfu JHMV, sacrificed at day 3 or 12 p.i., and brains were processed for FACS and viral titer. (**A**) Both the frequency and the total number of neutrophils infiltrating the brains of *CXCR2−/−* mice were significantly reduced compared to *CXCR2+/+* mice. However, no difference in (**B**) overall mortality to day 12 p.i., (**C**) viral load at days 3 and 12 p.i., or (**D**) EB extravasation into the brains were observed between infected *CXCR2+/+* and *CXCR2−/−* mice. (**E**) Qualitative PCR for CXCR1 transcripts revealed comparatively increased CXCR1 mRNA levels within neutrophils enriched from the bone marrow of *CXCR2−/−* compared to *CXCR2+/+* mice. Data in panels ***A***, ***C***, and ***D*** are representative of two independent experiments, n = 3–7 mice/time point. Data in panel ***B*** are a summation of two independent experiments, n = 9. Each group within panel ***E*** represents mRNA combined from two independent mice. *p<0.01 compared to *CXCR2+/+* mice.

We next utilized mice in which CXCR2 signaling was genetically silenced (*CXCR2−/−* mice) to further assess the functional role of CXCR2 in host defense in response to JHMV infection of the CNS. First, we sought to ensure that CXCR2 –deficient neutrophils were unresponsive to defined CXCR2 chemokine ligands. Our findings confirmed that PMNs isolated from the bone-marrow of *CXCR2−/−* mice did not express CXCR2 (**Figure 7A**), and, unlike *CXCR2+/+* PMN, *CXCR2−/−* PMN did not migrate *in vitro* in response to recombinant mouse CXCL1, CXCL2, or CXCL5 (**Figures 7 B–D**), consistent with previous reports [47],[48]. *CXCR2−/−* mice inoculated i.c. with JHMV exhibited an approximate 60% reduction (*p*<0.05) in PMN migration to the CNS compared to wildtype littermates at day 3 p.i. (**Figure 8A**). However, neutrophil infiltration into the CNS of *CXCR2−/−* mice was not completely eliminated, as previously observed following anti-CXCR2 treatment of JHMV-infected wildtype mice (**Figure 3A**). Correspondingly, there was no difference in mortality (**Figure 8B**) or brain viral titers (**Figure 8C**) between JHMV-infected *CXCR2−/−* mice and *CXCR2+/+* mice. Additionally, both *CXCR2−/−* and *CXCR2+/+* displayed similar amounts of Evans Blue extravasation into the brain at day 3 p.i. (**Figure 8D**). To assess potential compensatory mechanisms that may allow for *CXCR2−/−* neutrophils to enter the CNS, we evaluated expression of CXCR1, an alternate receptor for ELR+ chemokines [49], on neutrophils. Neutrophils isolated from the bone marrows of *CXCR2−/−* mice exhibited enriched levels of CXCR1 mRNA transcripts compared to *CXCR2+/+* neutrophils suggesting that, *in vivo*, this may be used as an alternate receptor for migration to the CNS (**Figure 8E**). In order to demonstrate that the CXCR2 anti-serum did not have any off – target effects, JHMV – infected CXCR2 – deficient and wildtype littermates were treated with anti-CXCR2 or NRS beginning at day -1 p.i. and PMN migration into the CNS at day 3 p.i. was determined. As shown in **Figure S4A**, anti-CXCR2 treatment did not affect neutrophil accumulation into the brains of *CXCR2−/−* animals, compared to *CXCR2−/−* mice receiving control serum. Similar numbers and frequencies of PMNs were also present within the CNS of knockout mice treated with either anti-CXCR2 antiserum or control serum (**Figure S4A**) compared to untreated *CXCR2−/−* mice infected with JHMV (**Figure 8A**). In contrast, anti-CXCR2 treatment of JHMV-infected *CXCR2+/+* mice reduced (>96%) PMN migration to the CNS (**Figure S4B**). These findings indicate that the CXCR2 blocking antibody is specific and suggest that genetic deletion of CXCR2 allows for compensatory mechanisms to emerge, such as utilization of CXCR1, that support PMN trafficking into the CNS in response to viral infection.

## Discussion

We have examined the role of ELR+ chemokines in host defense following infection of susceptible mice with the neurotropic coronavirus, JHMV. Early following JHMV infection, CXCR2-positive neutrophils are mobilized into the bloodstream and migrate to the CNS in response to elevated expression of the ELR+ chemokines CXCL1, CXCL2, and CXCL5. Early administration of a blocking antibody specific for CXCR2 to JHMV-infected mice reduced >95% of PMN migration into the CNS, and this corresponded with increased mortality and uncontrolled viral replication. Subsequent studies revealed that anti-CXCR2 treatment prevented PMN-mediated BBB permeabilization, associated with muted MMP-9 activity, and ultimately resulted in the impaired accumulation of virus-specific T cells within the CNS. These findings emphasize the importance of rapid neutrophil recruitment to the CNS in response to viral infection of the CNS to facilitate control of viral replication. Moreover, our findings highlight that the numbers of neutrophils recruited to the CNS is critical in defense as JHMV-infection of *CXCR2−/−* mice or delayed anti-CXCR2 treatment resulted in efficient control of viral replication, and this was associated with greater numbers of neutrophils within the CNS compared to mice treated prior to infection with blocking antibody. Collectively, our studies support and extend others highlighting the functional role of neutrophils in promoting vascular permeability in response to infection or injury to the CNS [25],[26],[50]. This is elegantly illustrated in the studies by McGavern and colleagues [50] that defined the importance of myelomonocytic cell recruitment to the CNS in response to LCMV infection with regards to contributing to fatal viral meningitis via promoting massive vascular injury. Importantly, interventional therapies targeting myeloid cell trafficking to the CNS during acute viral infection may offer a powerful approach to dampen neuroinflammation and decrease fatalities associated with viral encephalopathies.

In agreement with previous reports [51], we have also observed that CXCR2 signaling is important for neutrophil release from the bone marrow into the blood. Blocking CXCR2 dramatically reduced neutrophil mobilization into the bloodstream in response to JHMV infection. Notably, neutrophil entry into the blood was not completely inhibited, indicating that there may be additional signaling components that aid neutrophil release such as CXCL12 downregulation or G-CSF induction [51],[52]. CXCR2 neutralization also reduces circulating levels of neutrophils within uninfected mice (data not shown), suggesting that CXCR2 ligands contribute to both normal neutrophil homeostasis and emergency release following infection with a neurotropic virus. Our studies highlight a previously unappreciated functional role for ELR+ chemokines in host defense during viral-induced encephalomyelitis, rapidly recruiting PMNs into the blood with subsequent infiltration into the CNS, thus enhancing protection by contributing to the disruption of the BBB and facilitating anti-viral inflammatory T cell access. The protease MMP-9 is specific for the structural components of the BBB, including type IV collagen and laminin, and it is associated with BBB breakdown in a mouse model of cerebral ischemia [53]. Although MMP-9 is associated with the loss of BBB integrity following JHMV infection, it is likely that additional PMN-associated molecules including β_2_-integrin, azurocidin, and glutamate participate in BBB damage, as these components can also contribute to the disruption of endothelial cell adherens junctions [54]–[58]. Additionally, MMP-9 may also be contributing to the degradation of the parenchymal basement membranes and glia limitans that regulate leukocyte infiltration from the perivascular space into the parenchyma [59].

Infection of *CXCR2−/−* mice with JHMV did not recapitulate our observations with infected wildtype mice treated with CXCR2 antiserum. While neutrophil infiltration into the CNS was reduced by approximately 60% at day 3 p.i, CXCR2 – deficient mice experienced no deficits in survival, viral clearance, or BBB degradation. Our demonstration that PMN migration to the CNS of JHMV-infected *CXCR2−/−* mice was not altered following administration of anti-CXCR2 signaling emphasizes the specificity of this reagent and suggests that compensatory mechanisms allow for sufficient numbers of neutrophils to migrate to the CNS that enable BBB breakdown and infiltration of virus-specific T cells. However, it is notable that our observations are in contrast to previous reports utilizing *CXCR2−/−* mice in models of host defense following infection of the CNS. For example, Del Rio *et al*. [40] demonstrated elevated numbers of *T. gondii* cysts within the brains of *CXCR2−/−* mice, yet all of the immune defects occurred within the periphery, suggesting CXCR2-mediated protection occurs in a manner independent of leukocyte trafficking. Additionally, Kielian *et al*. [27] have shown that in a model of *S. aureus* experimental brain abscess neutrophil extravasation into the brain is impaired in *CXCR2−/−* mice, resulting in moderately increased bacterial burdens and pathology. However, the authors did not assess long-term bacterial clearance in the CXCR2 – deficient mice, nor quantitate total neutrophil or other immune cell infiltration that may have been impacted. Moreover, in experimental brain abscesses, neutrophils have direct anti-bacterial roles [60], while during JHMV infection, neutrophils are responsible for the permeabilization of the BBB [26], so functional differences of these cells in host defense may explain differences between the model systems. Our assessment of CXCR2 staining on neutrophils isolated within both the blood and brain of wildtype mice revealed that a small population of neutrophils was unreactive to CXCR2 antiserum. These findings argue for either transient receptor internalization following ligand binding [61] or alternative mechanisms of neutrophil attraction in the absence of CXCR2 signaling. Neutrophils have been reported to be chemotactic to cleaved complement products [48], leukotrienes [62], and other chemokines [63]–[65] *in vitro* and *in vivo*. Moreover, a functional murine homolog for CXCR1 has recently been identified and this may serve to mediate PMN trafficking to the CNS in response to JHMV infection [49]. Indeed, neutrophils enriched from the bone marrow of CXCR2 – deficient mice expressed noticeably increased transcripts specific for CXCR1, whereas transcripts were barely detectable within neutrophils from wild type mice, indicating that *CXCR2−/−* neutrophils may be compensating through increased expression of CXCR1. In addition, we have observed elevated numbers of PMNs residing within the CNS of *CXCR2−/−* mice in the absence of infection (data not shown), suggesting potentially highly dysregulated myeloid cells in these mice. The fact that these cells are present prior to JHMV-infection suggests the possibility that viral infection results in local activation that contributes to BBB breakdown and may account for the muted CD11b expression upon *CXCR2−/−* neutrophils within the CNS.

Recent evidence indicates that neutrophils are capable of influencing the generation of an adaptive immune response following infection. Neutrophil activation results in secretion of the cytokines IL-12/23 p40 that are associated with tailoring T cell-specific responses following antigenic challenge [66],[67]. Furthermore, neutrophils are capable of secreting chemokines such as CCL3 and CCL4 that also influence dendritic cell function, and thus they have been suggested to regulate T cell polarization via dendritic cell activation [68]. Additionally, the generation of T_H_1 immune reponses to a variety of pathogens is negatively impacted within neutropenic mice [41]–[46]. However, our findings clearly indicate that in the absence of CXCR2 signaling there are no deficiencies in either the frequencies or numbers of JHMV – specific T cells or T cell activation markers, regardless of whether mice were challenged within the CNS or peripherally.

Our results complement previous work that has highlighted the importance of ELR-positive chemokines and PMNs in promoting vascular permeability and subsequent immune cell infiltration into the CNS. CNS-specific transgenic expression of CXCL1 was associated with disruption of the BBB and neutrophil infiltration into the brain parenchyma [69]. Moreover, blocking CXCR2 signaling inhibits neutrophil-mediated BBB damage and dampens CNS inflammation in autoimmune demyelination and spinal cord injury [23]–[25]. Although the mechanisms associated with induction of ELR+ chemokines expression within the CNS of JHMV-infected mice have yet to be characterized, intracerebral administration or localized transgenic expression of IL-1β within the brain enhances CXCL1 expression, inducing neutrophil accumulation and subsequent BBB breakdown [70],[71]. Collectively, these findings have implications for other neurotropic viruses, including West Nile virus, as PMN represent an early and predominant inflammatory infiltrate in response to viral infection [72]. Therefore, understanding the signaling mechanisms governing inflammation in response to viral infection raises the possibility of selectively muting specific pathways associated with BBB breakdown and CNS inflammation which may have therapeutic benefits within the context of human neuroinflammatory diseases that arise in the apparent absence of an infectious trigger.

## Materials and Methods

### Virus and mice

Age-matched 5–6 week old C57BL/6 (H-2^b^, National Cancer Institute, Frederick, MD) or 5–9 week old *CXCR2−/−* (H-2^b^, Cleveland Clinic, OH) mice were infected intracerebrally (i.c.) with 500 plaque forming units (PFU) of JHMV strain J2.2v-1 in 30 µl of sterile HBSS. Control (sham) animals were injected with 30 µl of sterile saline alone. CXCR2 deficient mice [47] were originally backcrossed to C57BL/6 mice for 11 generations, and *CXCR2−/−* mice used for experimental purposes were obtained from heterozygous breeder pairs. All pups derived from breeders were genotyped as previously described [73]. Age and sex – matched littermate *CXCR2+/+* were used as controls for all experiments with *CXCR2−/−* mice. To assess the generation of JHMV – specific T cells, C57BL/6 mice were infected intraperitoneally (i.p.) with 2.5×10^5^ PFU of JHMV strain DM. For analysis of viral titers, one-half of each brain was homogenized and used for standard plaque assay on the DBT mouse astocytoma cell line [74] at the indicated days post-infection (p.i.). All experiments were approved by the University of California, Irvine Institutional Animal Care and Use Committee.

### Antibody production and administration

Rabbit polyclonal antiserum was generated to a 17-amino acid portion of the amino-terminus ligand binding domain of CXCR2 (MGEFKVDKFNIEDFFSG) [75]. In our hands, the CXCR2 antiserum specifically blocks CXCR2 dependent infiltration of neutrophils into the peritoneum of mice following thioglycollate irritation [76] and does not bind rabbit complement and deplete neutrophils *in vitro*. For *in vivo* neutralization during JHMV infection, 0.5 ml of anti-CXCR2 or control normal rabbit serum (NRS) was administered intraperitoneally (i.p.) on days −1, 1, 3, 5, 7, 9, and 11 p.i. or days 2, 4, 6, 8, and 10 p.i.

### Semi-quantitative real-time PCR

Total cDNA from the brains of sham and JHMV infected mice at days 1, 3, 7, 12, 15, 18, and 21 p.i. was generated as previously described [77]. Real-time Taqman analysis for HPRT, CXCR2, CXCL1, CXCL2, and CXCL5 was performed using a BioRad (Hercules, Ca) iCycler with previously described primers and probes [78],[79]. CXCR2 and CXCL1, −2, and −5 expression was normalized to HPRT. Probes were purchased from Integrated DNA Technologies (Coralville, IA), and primers were purchased from Invitrogen (Carlsbad, CA). iQ Supermix (BioRad) was used for all reactions. Assay conditions were as follows: a 4.5 min initial denaturation at 95°C, and 45 cycles of 30 sec at 95°C and 1 min at 58°C. Data were analyzed with BioRad iCycler iQ5 and quantified with the Relative Expression Software Tool [80].

### Flow cytometric analysis

Flow cytometry was performed as previously described [12],[17],[36]. Isolated cells were Fc blocked with anti-CD16/32 1∶200 (BD Biosciences, CA) and immunophenotyed with fluorescent antibodies (BD Biosciences) specific for the following cell surface markers: CD4 (L3T4), CD8b (53–5.8), CD8a (53–6.7), CD11b (M1/70), Ly6G (1A8), Ly6G/C (RB6–8C5), CD44 (IM7), CD25 (7D4), CD127 (A7R34, E Biosciences, Ca), CD45 (30-F11, E Biosciences), and F4/80 (CI:A3-1, Ab Direct, NC. Appropriate isotype antibodies were used for each antibody. For determination of viral specificity, isolated CNS cells, splenocytes, or cervical lymph node cells (CLN, isolated at day 7 p.i.) were either stained with H-2^b^-S510 tetramer (Beckman Coulter, CA) or stimulated *ex vivo* for 6 h with 5 µM of the immunodominant CD4 epitope M_133–147_ [38], immunodominant CD8 epitope S_510–518_ [39], or the subdominant CD8 epitope S_598–605_ [37] and GolgiStop (Cytofix/Cytoperm kit, BD Biosciences), and the production of IFNγ was determined by intracellular staining. Cells were Fc blocked with CD16/32 and stained with FITC or APC conjugated CD4, CD8a, or CD8b antibodies (BD Biosciences) before being fixed and permeablized with the Cytofix/Cytoperm kit and stained with PE conjugated IFNγ (XMG1.2, BD Biosciences). Appropriate isotype antibodies were used for each antibody. For CXCR2 staining isolated Fc – blocked cells were stained rabbit CXCR2 anti-serum (1∶1000) for 1 hour. NRS at 1∶1000 was used as a serum control. Cells were then washed and stained with APC – conjugated donkey anti-rabbit antibodies (1∶200, Jackson Immuno) for 30 min. Cells were run on a FACStar flow cytometer (BD Biosciences) and analyzed with FlowJo software (TreeStar, OR).

### Histology

Brains and spinal cords from 4% paraformaldehyde perfused mice were removed and fixed overnight in 4% paraformaldehyde at 4°C. Tissues were embedded in paraffin and stained with hematoxylin and eosin to determine the extent of inflammation.

### Immunostaining

Brains and spinal cords from 4% paraformaldehyde perfused mice were removed and fixed overnight in 4% paraformaldehyde at 4°C and cryoprotected in 20% sucrose. Tissue sections (7 µm) were fixed in 4% paraformaldehyde and blocked in 10% normal donkey serum, 0.3% Triton X 100. Immunostaining for CXCL1 and GFAP was performed serially using polyclonal goat anti-CXCL1 (2 ug/ml, R&D Systems, MN) and polyclonal chicken anti-GFAP (1∶500 Abcam, MA) overnight at 4°C. Cy-2 or Dylight 549 conjugated donkey secondary antibodies (1∶200, Jackson ImmunoResearch, PA) were used for visualization. Hoechst 33342 (Invitrogen) was used to stain nuclei prior to mounting coverslips.

### Evans Blue extravasation

At day 3 p.i., mice were injected with 200 µl of 2% (w/v) Evan's Blue in sterile PBS intraorbitally. Two hours later, brains and kidneys from PBS perfused mice were removed and homogenized in formamide (20 ml/g wet weight). Homogenates were incubated overnight, clarified by centrifugation, and assayed at 620 and 720 nm. CNS tissue turbidity was calculated [−log(OD_620_)  = 0.964(−log(OD_740_) −0.0357] and subtracted from the original A(620) [25],[81]. Relative permeability was calculated as the ratio of Evans blue extravasation (µg/ml per gram of tissue) of brain to kidney homogenates.

### MMP activity

Brains from PBS perfused mice were homogenized in 50 mM Tris-HCL 0.5% TritonX-100 pH 7.6 and clarified by centrifugation. 15 µg of homogenate were separated on polyacrylamide gels containing 1% gelatin (BioRad) in the absence of reducing agents. Gels were washed for 20 minutes and incubated in developing buffer (BioRad) for 2 days at 37°C. Gels were stained with Coomassie R-250 and destained in 10% acetic acid and 10% methanol. Quantification was performed with Image J software (v 1.42l, NIH) and expressed relative to sham activity.

### Neutrophil isolation and chemotaxis

Neutrophils were enriched from bone marrow as previously described [82]. Femurs and tibias were dissected from *CXCR2+/+* or *CXCR2−/−* mice. Marrow was flushed, and red blood cells lysed. Neutrophils were enriched using a 5 step percoll cushion: 45%, 50%, 55%, 62%, and 81%. Cells were collected from between the 81% and 62% percoll layers. Cells were washed, warmed at 37°C for 10 min, and 10^6^ cells were plated into the top well of a prewarmed 6.5 mm 5 µm transwell plate (Corning, NY #93421). Chemokine dilutions (Peprotech) at the indicated concentrations were made in the bottom well. Plates were incubated for 3 hours before cells in the bottom well were collected and counted. Data is presented as % input.

### CXCR1 PCR

Neutrophils were enriched from the bone marrows of *CXCR2+/+* and *CXCR2−/−* mice and cDNA was generated as previously described [77]. PCR was performed upon the generated cDNA with primers specific for CXCR1: forward 5′-GGGTGAAGCCACAACAGATT, reverse 5′-CGGTGTGTCAAAACCTCCTT and rpL32: forward 5′-AAGCGAAACTGGCGGAAACC, reverse 5′-CGTAGCCTGGCGTTGGGATT. Reaction conditions for CXCR1 were as follows: step 1, initial denaturation at 94°C for 3 min; step 2, denaturation at 94°C for 30 s; step 3, annealing at 58°C for 1 min; step 4, extension at 72°C for 30 s. Steps 2–4 were repeated 39 times for a total of 40 cycles. Reaction conditions for rpL32 were the same as above, except the annealing step was performed at 53°C. Sequencing of PCR amplicons confirmed primer specificity.

### Statistical analysis

All data is presented as average ± SEM. Statistically significant differences between groups of mice treated with anti-CXCR2 or NRS were assessed by one way ANOVA. Statistically significant differences between *CXCR2−/−* and *CXCR2+/+* mice were assessed by a two-tailed Mann Whitney U Test. Statistical significance on survival was performed with a Fisher Exact test. *P* values less than 0.05 were considered significant.

## Supporting Information

Figure S1 CXCR2 neutralization reduces levels of circulating blood neutrophils. C57BL/6 mice were infected i.c. with 500 pfu JHMV, administered CXCR2 antiserum or control normal rabbit serum (NRS) days -1 and +1 p.i. and sacrificed at day 3 p.i. to assess neutrophil levels within the blood. Blood was removed via the right ventricle of the heart and diluted into 1% BSA 5 mM EDTA in 1x PBS. Red blood cells were removed and the remaining cells were processed for FACS analysis with Ly6G/C (RB6-8C5) and CD11b specific antibodies. (A) Representative dot blots from experimental mice determining the frequency of neutrophils (Ly6G/C^high^/CD11b^+^) in blood. The frequency (average{plus minus}SEM) of gated cells is indicated. (B) CXCR2 antiserum treatment significantly reduced (*p*<0.05) but did not eliminate circulating neutrophils within the blood. Data in panels (A and B) are representative of two independent experiments with a minimum of 4 mice per group. The sham group did not receive any serum injections.
(0.54 MB PDF)

Figure S2 CXCR2 neutralization following JHMV infection does not affect mortality, viral burden, or T cell accumulation. C57BL/6 mice were infected i.c. with 500 PFU JHMV, administered CXCR2 antiserum or control normal rabbit serum (NRS) every other day from day 2 to 10 p.i., and sacrificed at the indicated days p.i. to assess viral burden and T cell infiltration within the brain. This treatment schedule did not alter (A) mortality (anti-CXCR2, n = 19; NRS, n = 23) or (B) the ability to control viral replication measured at day 12 p.i. (anti-CXCR2, n = 4–10; NRS, n = 4–8). Moreover, CD4+ (C) and CD8b+ (D) T cell accumulation within the brain was unaffected. (E) The accumulation of neutrophils between days 2 and 3 was however significantly reduced (*p*<0.01) following CXCR2 neutralization compared to control mice. Data in panels (C, D, & E) are representative of two independent experiments with a minimum of 4 mice per treatment group.
(0.87 MB PDF)

Figure S3 CXCR2 deficient mice generate JMHV - specific T cells. *CXCR2*−/− mice (n = 2) and *CXCR2*+/+ (n = 4) littermates controls were infected i.p. with 2.5×10^5^ PFU JHMV. Isolated splenocytes were collected 7 days later and or stimulated *ex vivo* for 6 hours with 5 µM of the immunodominant CD8 epitope S_510–518_ or the immunodominant CD4 epitope M_133–147_ and stained for IFN-γ production (A) or stained with S_510–518_ MHC-I tetramer (B).
(0.57 MB PDF)

Figure S4 CXCR2 antiserum does not alter neutrophil infiltration in CXCR2 - deficient mice. *CXCR2*+/+ and *CXCR2*−/− mice were infected i.c. with 500 pfu JHMV, treated with either 0.5 ml of anti-CXCR2 or control antisera on days -1 and +1 p.i., and sacrificed at day 3 p.i. to assess neutrophil infiltration into the brain. (A) CXCR2 antiserum did not affect neutrophil infiltration into the brains of *CXCR2*−/− mice, while (B) anti-CXCR2 completely prevented the infiltration of neutrophils into the brains of *CXCR2*+/+ mice. Representative FACS plots are shown with the average frequencies ± SEM, n = 3–4 for each treatment group.
(0.64 MB PDF)

## References

[1] Wang FI, Hinton DR, Gilmore W, Trousdale MD, Fleming JO (1992). Sequential infection of glial cells by the murine hepatitis virus JHM strain (MHV-4) leads to a characteristic distribution of demyelination.. Lab Invest.

[2] Lin MT, Stohlman SA, Hinton DR (1997). Mouse hepatitis virus is cleared from the central nervous systems of mice lacking perforin-mediated cytolysis.. J Virol.

[3] Parra B, Hinton DR, Marten NW, Bergmann CC, Lin MT (1999). IFN-gamma is required for viral clearance from central nervous system oligodendroglia.. J Immunol.

[4] Stohlman SA, Weiner LP (1981). Chronic central nervous system demyelination in mice after JHM virus infection.. Neurology.

[5] Marten NW, Stohlman SA, Atkinson RD, Hinton DR, Fleming JO (2000). Contributions of CD8+ T cells and viral spread to demyelinating disease.. J Immunol.

[6] Bergmann CC, Altman JD, Hinton D, Stohlman SA (1999). Inverted immunodominance and impaired cytolytic function of CD8+ T cells during viral persistence in the central nervous system.. J Immunol.

[7] Gonzalez JM, Bergmann CC, Ramakrishna C, Hinton DR, Atkinson R (2006). Inhibition of interferon-gamma signaling in oligodendroglia delays coronavirus clearance without altering demyelination.. Am J Pathol.

[8] Cheever FS, Daniels JB, Pappenheimer AM, Bailey OT (1949). A murine virus (JHM) causing disseminated encephalomyelitis with extensive destruction of myelin.. Journal of Exerimental Medicine.

[9] Perlman SR, Lane TE, Buchmeier MJ, Cunningham MW, Fujinami RS (1999). Coronaviruses: Hepatitis, peritonitis, and central nervous system disease.. Effects of Microbes on the Immune System.

[10] Glass WG, Liu MT, Kuziel WA, Lane TE (2001). Reduced macrophage infiltration and demyelination in mice lacking the chemokine receptor CCR5 following infection with a neurotropic coronavirus.. Virology.

[11] Lane TE, Asensio VC, Yu N, Paoletti AD, Campbell IL (1998). Dynamic regulation of alpha- and beta-chemokine expression in the central nervous system during mouse hepatitis virus-induced demyelinating disease.. J Immunol.

[12] Lane TE, Liu MT, Chen BP, Asensio VC, Samawi RM (2000). A central role for CD4(+) T cells and RANTES in virus-induced central nervous system inflammation and demyelination.. J Virol.

[13] Liu MT, Armstrong D, Hamilton TA, Lane TE (2001). Expression of Mig (monokine induced by interferon-gamma) is important in T lymphocyte recruitment and host defense following viral infection of the central nervous system.. J Immunol.

[14] Liu MT, Chen BP, Oertel P, Buchmeier MJ, Armstrong D (2000). The T cell chemoattractant IFN-inducible protein 10 is essential in host defense against viral-induced neurologic disease.. J Immunol.

[15] Glass WG, Hickey MJ, Hardison JL, Liu MT, Manning JE (2004). Antibody targeting of the CC chemokine ligand 5 results in diminished leukocyte infiltration into the central nervous system and reduced neurologic disease in a viral model of multiple sclerosis.. J Immunol.

[16] Liu MT, Keirstead HS, Lane TE (2001). Neutralization of the chemokine CXCL10 reduces inflammatory cell invasion and demyelination and improves neurological function in a viral model of multiple sclerosis.. J Immunol.

[17] Stiles LN, Hosking MP, Edwards RA, Strieter RM, Lane TE (2006). Differential roles for CXCR3 in CD4+ and CD8+ T cell trafficking following viral infection of the CNS.. Eur J Immunol.

[18] Scott EP, Branigan PJ, Del Vecchio AM, Weiss SR (2008). Chemokine expression during mouse-hepatitis-virus-induced encephalitis: contributions of the spike and background genes.. J Neurovirol.

[19] Zhou J, Stohlman SA, Atkinson R, Hinton DR, Marten NW (2002). Matrix metalloproteinase expression correlates with virulence following neurotropic mouse hepatitis virus infection.. J Virol.

[20] Moser B, Clark-Lewis I, Zwahlen R, Baggiolini M (1990). Neutrophil-activating properties of the melanoma growth-stimulatory activity.. J Exp Med.

[21] Schumacher C, Clark-Lewis I, Baggiolini M, Moser B (1992). High- and low-affinity binding of GRO alpha and neutrophil-activating peptide 2 to interleukin 8 receptors on human neutrophils.. Proc Natl Acad Sci U S A.

[22] Wolpe SD, Sherry B, Juers D, Davatelis G, Yurt RW (1989). Identification and characterization of macrophage inflammatory protein 2.. Proc Natl Acad Sci U S A.

[23] Gorio A, Madaschi L, Zadra G, Marfia G, Cavalieri B (2007). Reparixin, an inhibitor of CXCR2 function, attenuates inflammatory responses and promotes recovery of function after traumatic lesion to the spinal cord.. J Pharmacol Exp Ther.

[24] Tonai T, Shiba K, Taketani Y, Ohmoto Y, Murata K (2001). A neutrophil elastase inhibitor (ONO-5046) reduces neurologic damage after spinal cord injury in rats.. J Neurochem.

[25] Carlson T, Kroenke M, Rao P, Lane TE, Segal B (2008). The Th17-ELR+ CXC chemokine pathway is essential for the development of central nervous system autoimmune disease.. J Exp Med.

[26] Zhou J, Stohlman SA, Hinton DR, Marten NW (2003). Neutrophils promote mononuclear cell infiltration during viral-induced encephalitis.. J Immunol.

[27] Kielian T, Barry B, Hickey WF (2001). CXC chemokine receptor-2 ligands are required for neutrophil-mediated host defense in experimental brain abscesses.. J Immunol.

[28] Belperio JA, Keane MP, Burdick MD, Gomperts BN, Xue YY (2005). CXCR2/CXCR2 ligand biology during lung transplant ischemia-reperfusion injury.. J Immunol.

[29] Londhe VA, Belperio JA, Keane MP, Burdick MD, Xue YY (2005). CXCR2/CXCR2 ligand biological axis impairs alveologenesis during dsRNA-induced lung inflammation in mice.. Pediatr Res.

[30] Londhe VA, Belperio JA, Keane MP, Burdick MD, Xue YY (2005). CXCR2 is critical for dsRNA-induced lung injury: relevance to viral lung infection.. J Inflamm (Lond).

[31] Strieter RM, Keane MP, Burdick MD, Sakkour A, Murray LA (2005). The role of CXCR2/CXCR2 ligands in acute lung injury.. Curr Drug Targets Inflamm Allergy.

[32] Wareing MD, Shea AL, Inglis CA, Dias PB, Sarawar SR (2007). CXCR2 is required for neutrophil recruitment to the lung during influenza virus infection, but is not essential for viral clearance.. Viral Immunol.

[33] Omari KM, John G, Lango R, Raine CS (2006). Role for CXCR2 and CXCL1 on glia in multiple sclerosis.. Glia.

[34] Rubio N, Sanz-Rodriguez F (2007). Induction of the CXCL1 (KC) chemokine in mouse astrocytes by infection with the murine encephalomyelitis virus of Theiler.. Virology.

[35] Romagnani P, Lasagni L, Annunziato F, Serio M, Romagnani S (2004). CXC chemokines: the regulatory link between inflammation and angiogenesis.. Trends Immunol.

[36] Trifilo MJ, Lane TE (2004). The CC chemokine ligand 3 regulates CD11c+CD11b+CD8alpha- dendritic cell maturation and activation following viral infection of the central nervous system: implications for a role in T cell activation.. Virology.

[37] Castro RF, Perlman S (1995). CD8+ T-cell epitopes within the surface glycoprotein of a neurotropic coronavirus and correlation with pathogenicity.. J Virol.

[38] Xue S, Jaszewski A, Perlman S (1995). Identification of a CD4+ T cell epitope within the M protein of a neurotropic coronavirus.. Virology.

[39] Bergmann CC, Yao Q, Lin M, Stohlman SA (1996). The JHM strain of mouse hepatitis virus induces a spike protein-specific Db-restricted cytotoxic T cell response.. J Gen Virol.

[40] Del Rio L, Bennouna S, Salinas J, Denkers EY (2001). CXCR2 deficiency confers impaired neutrophil recruitment and increased susceptibility during Toxoplasma gondii infection.. J Immunol.

[41] Romani L, Mencacci A, Cenci E, Del Sero G, Bistoni F (1997). An immunoregulatory role for neutrophils in CD4+ T helper subset selection in mice with candidiasis.. J Immunol.

[42] Appelberg R, Castro AG, Silva MT (1994). Neutrophils as effector cells of T-cell-mediated, acquired immunity in murine listeriosis.. Immunology.

[43] Pedrosa J, Saunders BM, Appelberg R, Orme IM, Silva MT (2000). Neutrophils play a protective nonphagocytic role in systemic Mycobacterium tuberculosis infection of mice.. Infect Immun.

[44] Ismail HF, Fick P, Zhang J, Lynch RG, Berg DJ (2003). Depletion of neutrophils in IL-10(-/-) mice delays clearance of gastric Helicobacter infection and decreases the Th1 immune response to Helicobacter.. J Immunol.

[45] McFarlane E, Perez C, Charmoy M, Allenbach C, Carter KC (2008). Neutrophils contribute to development of a protective immune response during onset of infection with Leishmania donovani.. Infect Immun.

[46] Tateda K, Moore TA, Deng JC, Newstead MW, Zeng X (2001). Early recruitment of neutrophils determines subsequent T1/T2 host responses in a murine model of Legionella pneumophila pneumonia.. J Immunol.

[47] Cacalano G, Lee J, Kikly K, Ryan AM, Pitts-Meek S (1994). Neutrophil and B cell expansion in mice that lack the murine IL-8 receptor homolog.. Science.

[48] Lee J, Cacalano G, Camerato T, Toy K, Moore MW (1995). Chemokine binding and activities mediated by the mouse IL-8 receptor.. J Immunol.

[49] Fan X, Patera AC, Pong-Kennedy A, Deno G, Gonsiorek W (2007). Murine CXCR1 is a functional receptor for GCP-2/CXCL6 and interleukin-8/CXCL8.. J Biol Chem.

[50] Kim JV, Kang SS, Dustin ML, McGavern DB (2009). Myelomonocytic cell recruitment causes fatal CNS vascular injury during acute viral meningitis.. Nature.

[51] Wengner AM, Pitchford SC, Furze RC, Rankin SM (2008). The coordinated action of G-CSF and ELR + CXC chemokines in neutrophil mobilization during acute inflammation.. Blood.

[52] Martin C, Burdon PC, Bridger G, Gutierrez-Ramos JC, Williams TJ (2003). Chemokines acting via CXCR2 and CXCR4 control the release of neutrophils from the bone marrow and their return following senescence.. Immunity.

[53] Asahi M, Wang X, Mori T, Sumii T, Jung JC (2001). Effects of matrix metalloproteinase-9 gene knock-out on the proteolysis of blood-brain barrier and white matter components after cerebral ischemia.. J Neurosci.

[54] Collard CD, Park KA, Montalto MC, Alapati S, Buras JA (2002). Neutrophil-derived glutamate regulates vascular endothelial barrier function.. J Biol Chem.

[55] Del Maschio A, Zanetti A, Corada M, Rival Y, Ruco L (1996). Polymorphonuclear leukocyte adhesion triggers the disorganization of endothelial cell-to-cell adherens junctions.. J Cell Biol.

[56] Gautam N, Herwald H, Hedqvist P, Lindbom L (2000). Signaling via beta(2) integrins triggers neutrophil-dependent alteration in endothelial barrier function.. J Exp Med.

[57] Ionescu CV, Cepinskas G, Savickiene J, Sandig M, Kvietys PR (2003). Neutrophils induce sequential focal changes in endothelial adherens junction components: role of elastase.. Microcirculation.

[58] Watorek W (2003). Azurocidin–inactive serine proteinase homolog acting as a multifunctional inflammatory mediator.. Acta Biochim Pol.

[59] Agrawal S, Anderson P, Durbeej M, van Rooijen N, Ivars F (2006). Dystroglycan is selectively cleaved at the parenchymal basement membrane at sites of leukocyte extravasation in experimental autoimmune encephalomyelitis.. J Exp Med.

[60] Kielian T (2004). Immunopathogenesis of brain abscess.. J Neuroinflammation.

[61] Dal-Secco D, Cunha TM, Freitas A, Alves-Filho JC, Souto FO (2008). Hydrogen sulfide augments neutrophil migration through enhancement of adhesion molecule expression and prevention of CXCR2 internalization: role of ATP-sensitive potassium channels.. J Immunol.

[62] Palmblad J, Malmsten CL, Uden AM, Radmark O, Engstedt L (1981). Leukotriene B4 is a potent and stereospecific stimulator of neutrophil chemotaxis and adherence.. Blood.

[63] Wu L, Ruffing N, Shi X, Newman W, Soler D (1996). Discrete steps in binding and signaling of interleukin-8 with its receptor.. J Biol Chem.

[64] Furuichi K, Gao JL, Horuk R, Wada T, Kaneko S (2008). Chemokine receptor CCR1 regulates inflammatory cell infiltration after renal ischemia-reperfusion injury.. J Immunol.

[65] Ramos CD, Canetti C, Souto JT, Silva JS, Hogaboam CM (2005). MIP-1alpha[CCL3] acting on the CCR1 receptor mediates neutrophil migration in immune inflammation via sequential release of TNF-alpha and LTB4.. J Leukoc Biol.

[66] Bliss SK, Butcher BA, Denkers EY (2000). Rapid recruitment of neutrophils containing prestored IL-12 during microbial infection.. J Immunol.

[67] Romani L, Mencacci A, Cenci E, Spaccapelo R, Del Sero G (1997). Neutrophil production of IL-12 and IL-10 in candidiasis and efficacy of IL-12 therapy in neutropenic mice.. J Immunol.

[68] van Gisbergen KP, Geijtenbeek TB, van Kooyk Y (2005). Close encounters of neutrophils and DCs.. Trends Immunol.

[69] Tani M, Fuentes ME, Peterson JW, Trapp BD, Durham SK (1996). Neutrophil infiltration, glial reaction, and neurological disease in transgenic mice expressing the chemokine N51/KC in oligodendrocytes.. J Clin Invest.

[70] Anthony D, Dempster R, Fearn S, Clements J, Wells G (1998). CXC chemokines generate age-related increases in neutrophil-mediated brain inflammation and blood-brain barrier breakdown.. Curr Biol.

[71] Shaftel SS, Carlson TJ, Olschowka JA, Kyrkanides S, Matousek SB (2007). Chronic interleukin-1beta expression in mouse brain leads to leukocyte infiltration and neutrophil-independent blood brain barrier permeability without overt neurodegeneration.. J Neurosci.

[72] Davis LE, DeBiasi R, Goade DE, Haaland KY, Harrington JA (2006). West Nile virus neuroinvasive disease.. Ann Neurol.

[73] Tsai HH, Frost E, To V, Robinson S, Ffrench-Constant C (2002). The chemokine receptor CXCR2 controls positioning of oligodendrocyte precursors in developing spinal cord by arresting their migration.. Cell.

[74] Hirano N, Murakami T, Fujiwara K, Matsumoto M (1978). Utility of mouse cell line DBT for propagation and assay of mouse hepatitis virus.. Jpn J Exp Med.

[75] Mehrad B, Strieter RM, Moore TA, Tsai WC, Lira SA (1999). CXC chemokine receptor-2 ligands are necessary components of neutrophil-mediated host defense in invasive pulmonary aspergillosis.. J Immunol.

[76] Call DR, Nemzek JA, Ebong SJ, Bolgos GL, Newcomb DE (2001). Ratio of local to systemic chemokine concentrations regulates neutrophil recruitment.. Am J Pathol.

[77] Walsh KB, Lanier LL, Lane TE (2008). NKG2D receptor signaling enhances cytolytic activity by virus-specific CD8+ T cells: evidence for a protective role in virus-induced encephalitis.. J Virol.

[78] Carollo M, Hogaboam CM, Kunkel SL, Delaney S, Christie MI (2001). Analysis of the temporal expression of chemokines and chemokine receptors during experimental granulomatous inflammation: role and expression of MIP-1alpha and MCP-1.. Br J Pharmacol.

[79] Ogasawara K, Hamerman JA, Hsin H, Chikuma S, Bour-Jordan H (2003). Impairment of NK cell function by NKG2D modulation in NOD mice.. Immunity.

[80] Pfaffl MW (2001). A new mathematical model for relative quantification in real-time RT-PCR.. Nucleic Acids Res.

[81] Warnick RE, Fike JR, Chan PH, Anderson DK, Ross GY (1995). Measurement of vascular permeability in spinal cord using Evans Blue spectrophotometry and correction for turbidity.. J Neurosci Methods.

[82] Lowell CA, Fumagalli L, Berton G (1996). Deficiency of Src family kinases p59/61hck and p58c-fgr results in defective adhesion-dependent neutrophil functions.. J Cell Biol.

