# Supplementary figures and images for: A Protective Role for ELR+ Chemokines during Acute Viral Encephalomyelitis
Source: PLoS Pathog. 2009 Nov 6;5(11):e1000648. doi: 10.1371/journal.ppat.1000648 (PMC2766051; doi:10.1371/journal.ppat.1000648)

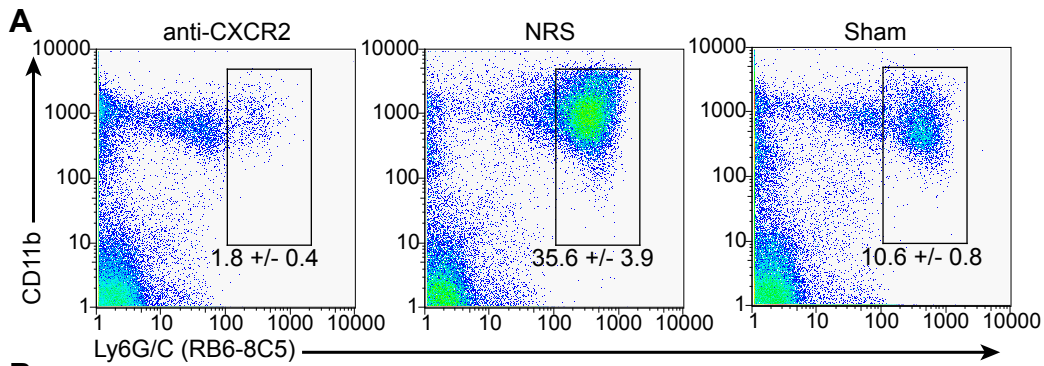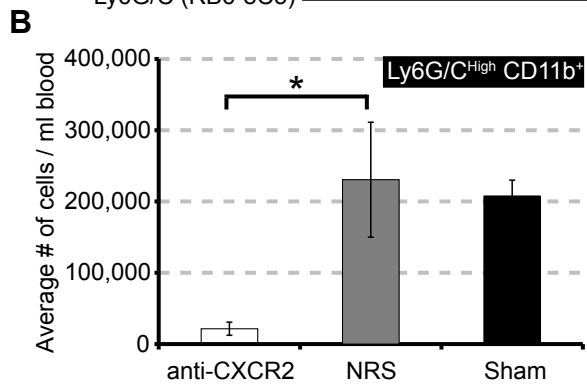

Supplement: Figure S1 — CXCR2 neutralization reduces levels of circulating blood neutrophils. C57BL/6 mice were infected i.c. with 500 pfu JHMV, administered CXCR2 antiserum or control normal rabbit serum (NRS) days -1 and +1 p.i. and sacrificed at day 3 p.i. to assess neutrophil levels within the blood. Blood was removed via the right ventricle of the heart and diluted into 1% BSA 5 mM EDTA in 1x PBS. Red blood cells were removed and the remaining cells were processed for FACS analysis with Ly6G/C (RB6-8C5) and CD11b specific antibodies. (A) Representative dot blots from experimental mice determining the frequency of neutrophils (Ly6G/Chigh/CD11b+) in blood. The frequency (average{plus minus}SEM) of gated cells is indicated. (B) CXCR2 antiserum treatment significantly reduced (p<0.05) but did not eliminate circulating neutrophils within the blood. Data in panels (A and B) are representative of two independent experiments with a minimum of 4 mice per group. The sham group did not receive any serum injections. (0.54 MB PDF) [file ppat.1000648.s001.pdf]

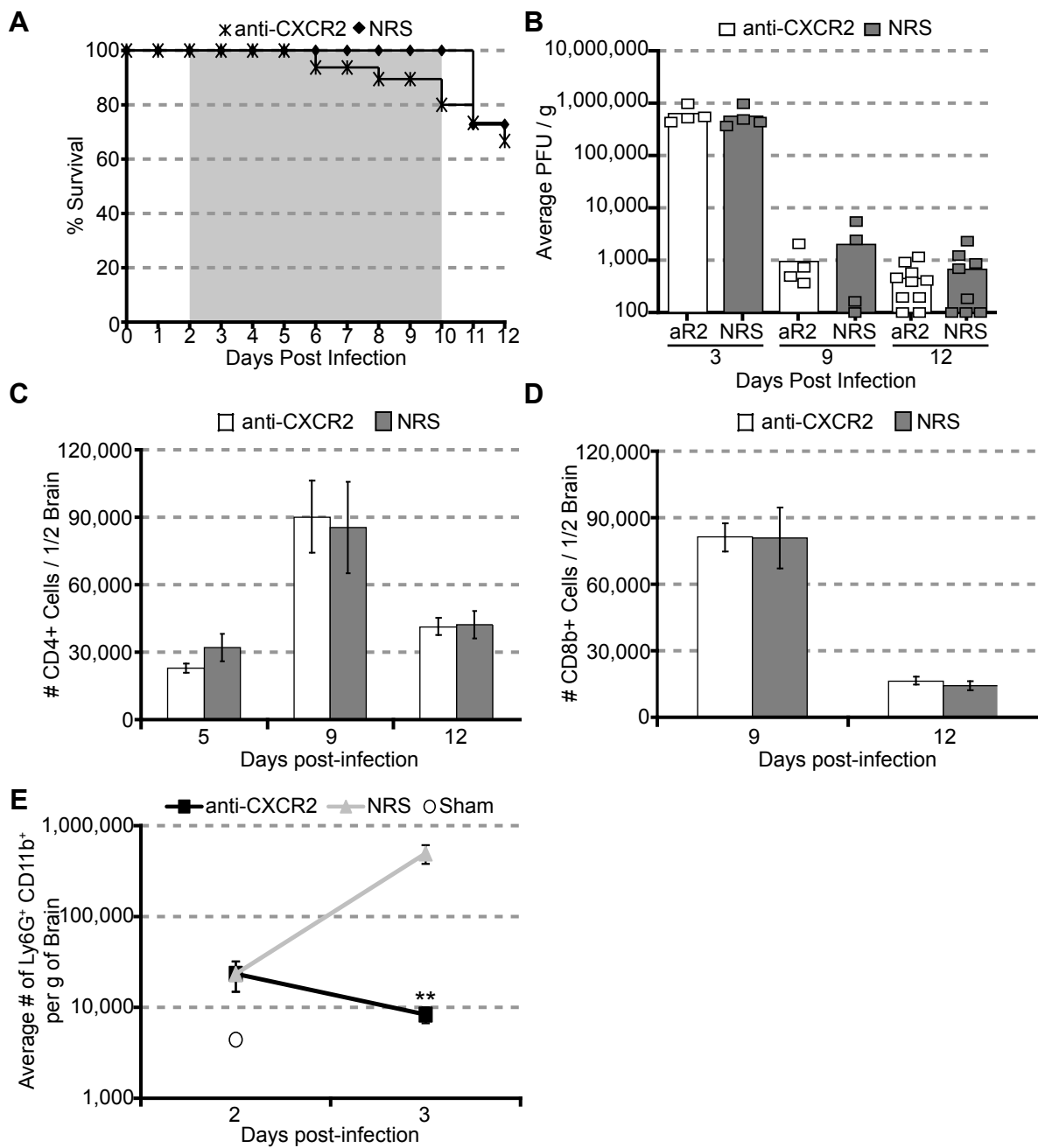

Supplement: Figure S2 — CXCR2 neutralization following JHMV infection does not affect mortality, viral burden, or T cell accumulation. C57BL/6 mice were infected i.c. with 500 PFU JHMV, administered CXCR2 antiserum or control normal rabbit serum (NRS) every other day from day 2 to 10 p.i., and sacrificed at the indicated days p.i. to assess viral burden and T cell infiltration within the brain. This treatment schedule did not alter (A) mortality (anti-CXCR2, n = 19; NRS, n = 23) or (B) the ability to control viral replication measured at day 12 p.i. (anti-CXCR2, n = 4–10; NRS, n = 4–8). Moreover, CD4+ (C) and CD8b+ (D) T cell accumulation within the brain was unaffected. (E) The accumulation of neutrophils between days 2 and 3 was however significantly reduced (p<0.01) following CXCR2 neutralization compared to control mice. Data in panels (C, D, & E) are representative of two independent experiments with a minimum of 4 mice per treatment group. (0.87 MB PDF) [file ppat.1000648.s002.pdf]

**A**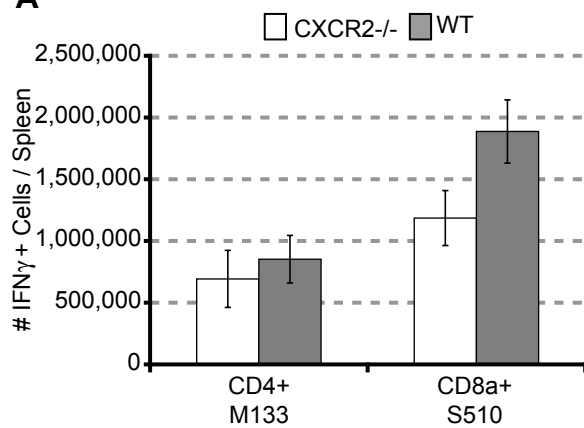**B**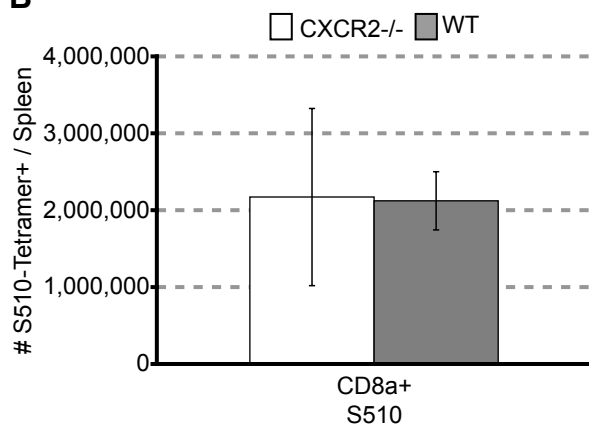

Supplement: Figure S3 — CXCR2 deficient mice generate JMHV - specific T cells. CXCR2−/− mice (n = 2) and CXCR2+/+ (n = 4) littermates controls were infected i.p. with 2.5×10^5^ PFU JHMV. Isolated splenocytes were collected 7 days later and or stimulated ex vivo for 6 hours with 5 µM of the immunodominant CD8 epitope S510–518 or the immunodominant CD4 epitope M133–147 and stained for IFN-γ production (A) or stained with S510–518 MHC-I tetramer (B). (0.57 MB PDF) [file ppat.1000648.s003.pdf]

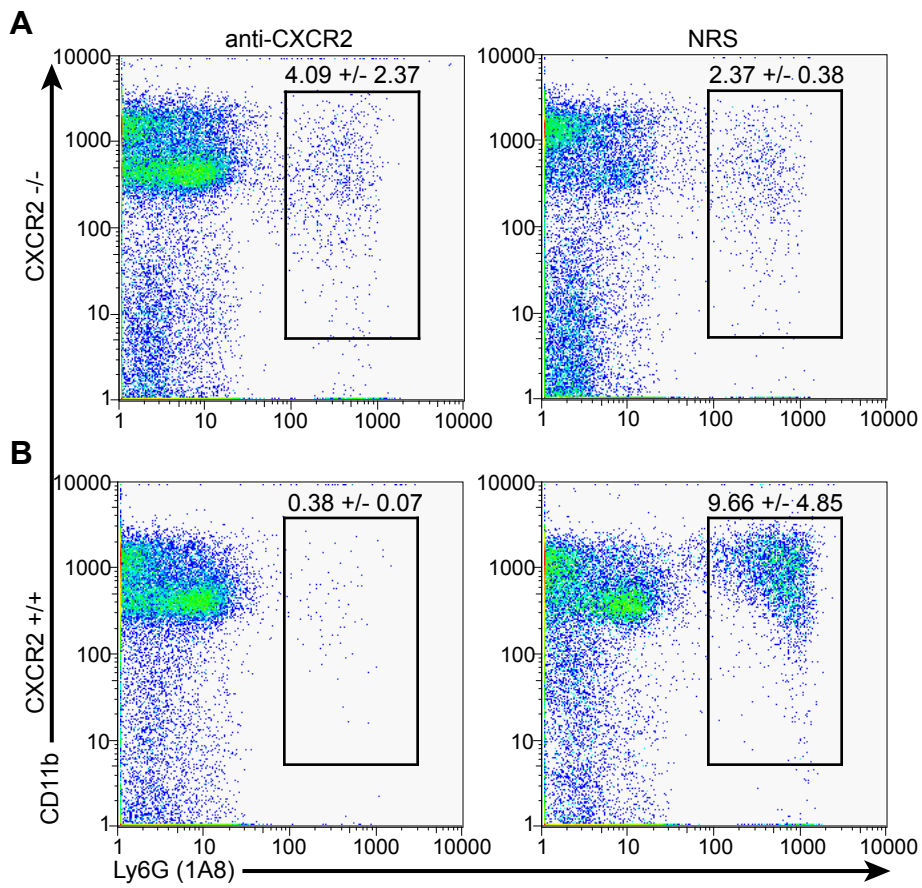

Supplement: Figure S4 — CXCR2 antiserum does not alter neutrophil infiltration in CXCR2 - deficient mice. CXCR2+/+ and CXCR2−/− mice were infected i.c. with 500 pfu JHMV, treated with either 0.5 ml of anti-CXCR2 or control antisera on days -1 and +1 p.i., and sacrificed at day 3 p.i. to assess neutrophil infiltration into the brain. (A) CXCR2 antiserum did not affect neutrophil infiltration into the brains of CXCR2−/− mice, while (B) anti-CXCR2 completely prevented the infiltration of neutrophils into the brains of CXCR2+/+ mice. Representative FACS plots are shown with the average frequencies ± SEM, n = 3–4 for each treatment group. (0.64 MB PDF) [file ppat.1000648.s004.pdf]
